# Supplementary material for: Cholesterol synthesis pathway genes in prostate cancer are transcriptionally downregulated when tissue confounding is minimized
Source: BMC Cancer. 2018 Apr 27;18:478. doi: 10.1186/s12885-018-4373-y (PMC5922022; doi:10.1186/s12885-018-4373-y)
Supplement: Supplementary file 1 — Figure S1. Quality assessment of the seven patient cohorts used in this study. Figure S2. Percentage of stroma genes shared by comparing genes identified by our described selection procedure to genes identified by a naïve approach using only Pearson correlation to normal stroma content over all samples. Figure S3. GSEA score correlations using the stroma gene sets from Bertilsson and Chen independently for samples in all cohorts. Figure S4. Significantly differentially expressed genes (prostate cancer compared to normal) related to the cholesterol synthesis pathway calculated for each of the five patient cohorts having both prostate cancer and normal samples, as well as the meta-study for the seven-study-cohort. Figure S5. Significantly differentially expressed genes (prostate cancer compared to normal) related to regulation, uptake, efflux and transport of cholesterol, calculated for each of the five patient cohorts containing prostate cancer and normal samples, as well as the meta-study for the seven-study-cohort. Figure S6. Significantly differentially expressed genes (prostate cancer compared to normal) in the Bertilsson cohort after samples from patients reported to have taken statin prior to surgery have been removed (in total 26 samples, 18 cancer and 8 normal). Table S1. GSEA score stabilities using various numbers of the top ranked stroma genes in the gene-sets from Bertilsson and Chen. Table S2. Number of cancer/normal samples and average Gleason score in balanced and unbalanced datasets from all cohorts. (PDF 1575 kb) [file 12885_2018_4373_MOESM1_ESM.pdf]

Cholesterol Synthesis Pathway Genes in Prostate Cancer are consistently downregulated

Morten Beck Rye

Helena Bertilsson

Maria K. Andersen

Kjersti Rise

Tone F. Bathen

Finn Drabløs

May-Britt Tessem

Additional file 1: Figure S1-Figure S6 and Table S1-Table S2.

Figure S1: Quality assessment of the seven patient cohorts used in this study. The average Pearson correlation between all genes in four previously published and validated ERG-fusion signatures (1, 2) was calculated independently for prostate cancer and normal samples in each cohort. Since ERG-fusion is an established feature present in about 50% of all prostate cancer, it would be expected to see an improved correlation between genes related to this feature in the cancer samples compared to the normal samples. All five cohorts with both cancer and normal samples showed improved correlation in the cancer samples. Of the two cohorts with only cancer samples, the cohort from *Erho* showed comparable correlation to the other prostate cancer cohorts, while the cohort from *Sboner* displayed a somewhat lower correlation, but still above the correlation in most of the normal samples.

Figure S2: Percentage of stroma genes shared by comparing genes identified by our described selection procedure to genes identified by a naïve approach using only Pearson correlation to normal stroma content over all samples. The percentage was calculated selecting the N most highly ranked genes in each selection for various N. The percentage overlap of genes using the two procedures is substantial.

Figure S3: GSEA score correlations using the stroma gene sets from *Bertilsson* and *Chen* independently for samples in all cohorts. For the *five-dataset-cohort*, scores calculated from the 9527 shared genes between these five cohorts were compared, while GSEA scores in the *Sboner* and *Erho* cohorts were calculated from the 4804 genes shared by all seven cohorts for the comparison.

Figure S4: Significantly differentially expressed genes (prostate cancer compared to normal) related to the cholesterol synthesis pathway calculated for each of the five patient cohorts having both prostate cancer and normal samples, as well as the meta-study for the *seven-study-cohort*. For the *seven-study-cohort* differential expression was calculated using the Mann-Whitney-Wilcoxon test using ranked genes for each sample centered for each cohort. The figure shows –log10 p-values multiplied by 1 for upregulated genes, and -1 for downregulated genes. Genes not found in a cohort are marked in red. All p-values were corrected for multiple testing using the complete list of all measured genes in each cohort.

Figure S5: Significantly differentially expressed genes (prostate cancer compared to normal) related to regulation, uptake, efflux and transport of cholesterol, calculated for each of the five patient cohorts containing prostate cancer and normal samples, as well as the meta-study for the *seven-study-cohort*. For the *seven-study-cohort* differential expression was calculated using the Mann-Whitney-Wilcoxon test using ranked genes for each sample centered for each cohort. The figure shows –log10 p-values multiplied by 1 for upregulated genes, and -1 for downregulated genes. Genes not found in a cohort are marked in red. All p-values were corrected for multiple testing using the complete list of all measured genes in each cohort.

Figure S6: Significantly differentially expressed genes (prostate cancer compared to normal) in the *Bertilsson* cohort after samples from patients reported to have taken statin prior to surgery have been removed (in total 26 samples, 18 cancer and 8 normal). Removal of samples did not affect the overall transcriptional pattern in the cholesterol pathway and regulation. All p-values were corrected for multiple testing using the complete list of 14 149 measured unique genes in the *Bertilsson* cohort.

Table S1: GSEA score stabilities using various numbers of the top ranked stroma genes in the gene-sets from *Bertilsson* and *Chen*.

Table S2: Number of cancer/normal samples and average Gleason score in *balanced* and *unbalanced* datasets from all cohorts.

1. Markert EK, Mizuno H, Vazquez A, Levine AJ. Molecular classification of prostate cancer using curated expression signatures. Proc Natl Acad Sci U S A. 2011;108:21276-81.
2. Rye MB, Bertilsson H, Drablos F, Angelsen A, Bathen TF, Tessem MB. Gene signatures ESC, MYC and ERG-fusion are early markers of a potentially dangerous subtype of prostate cancer. BMC medical genomics. 2014;7:50

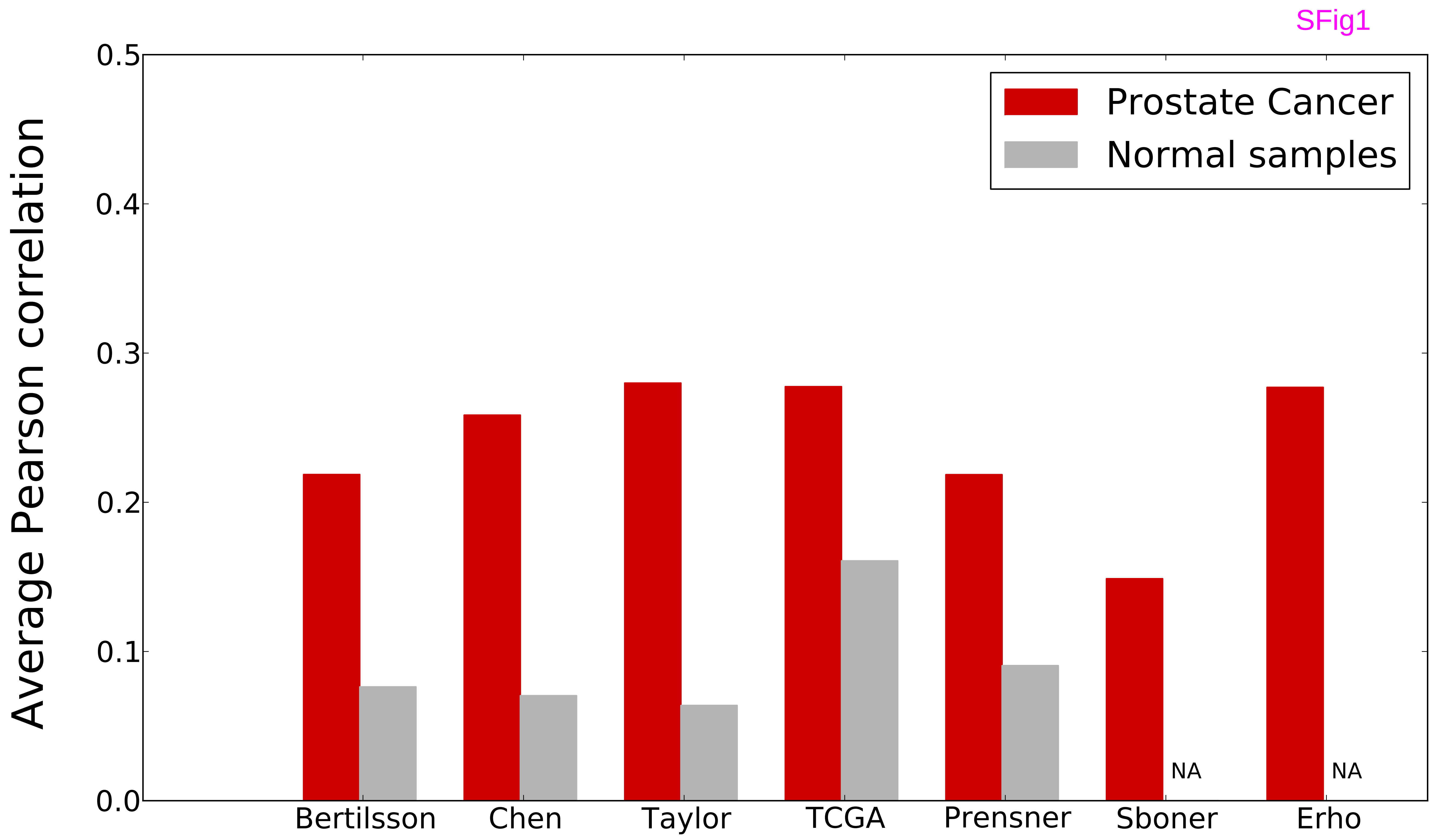

SFig1
Average Pearson correlation
0.5
0.4
0.3
0.2
0.1
0.0
Prostate Cancer
Normal samples
NA
NA
Bertilsson
Chen
Taylor
TCGA
Prensner
Sboner
Erho

SFig2

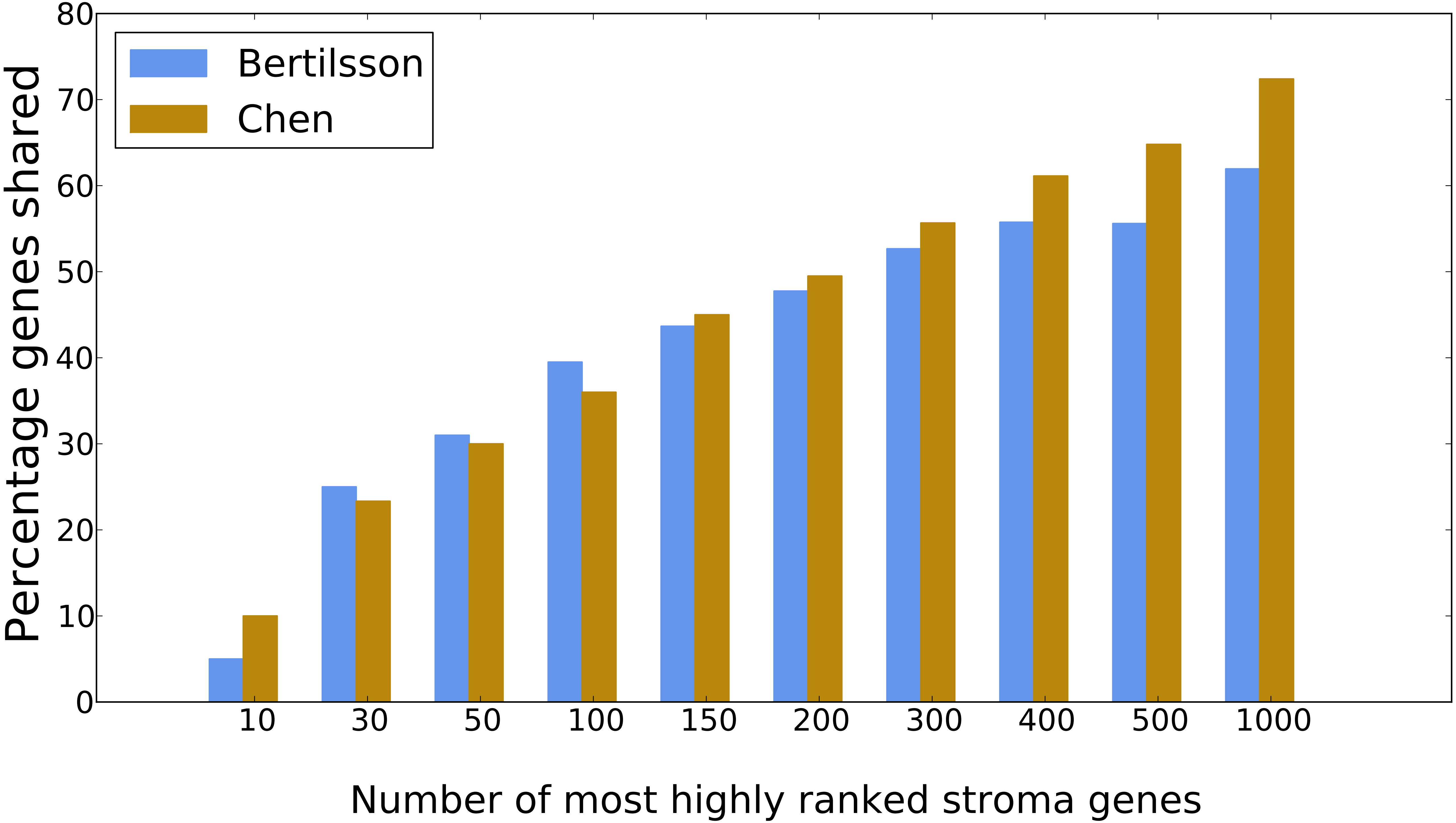

Bertilsson
Chen
Percentage genes shared
0
10
20
30
40
50
60
70
80
10
30
50
100
150
200
300
400
500
1000
Number of most highly ranked stroma genes

SFig3

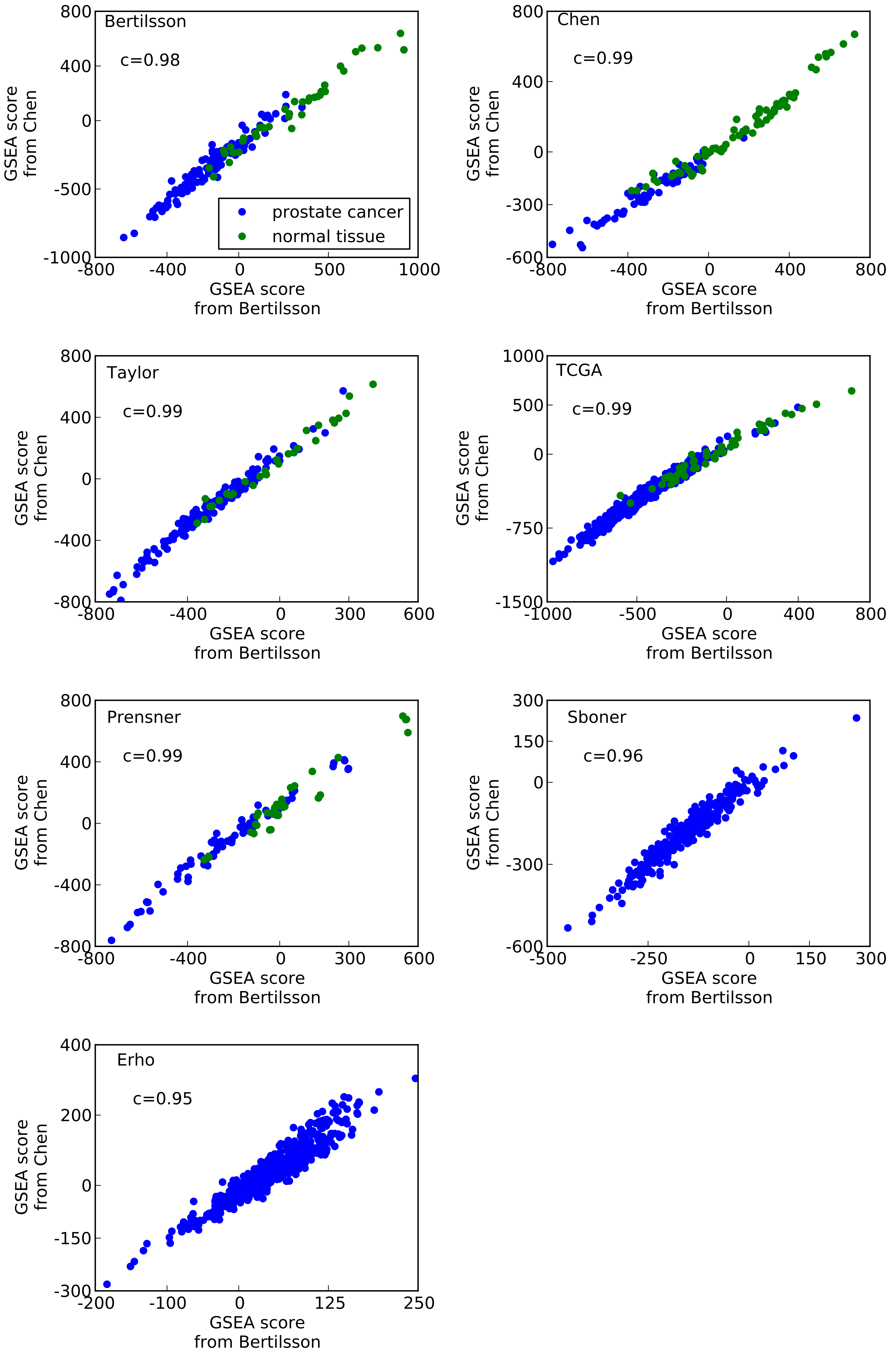
Bertilsson
c=0.98
prostate cancer
normal tissue
GSEA score from Chen
GSEA score from Bertilsson
Chen
c=0.99
Taylor
c=0.99
TCGA
c=0.99
Prensner
c=0.99
Sboner
c=0.96
Erho
c=0.95

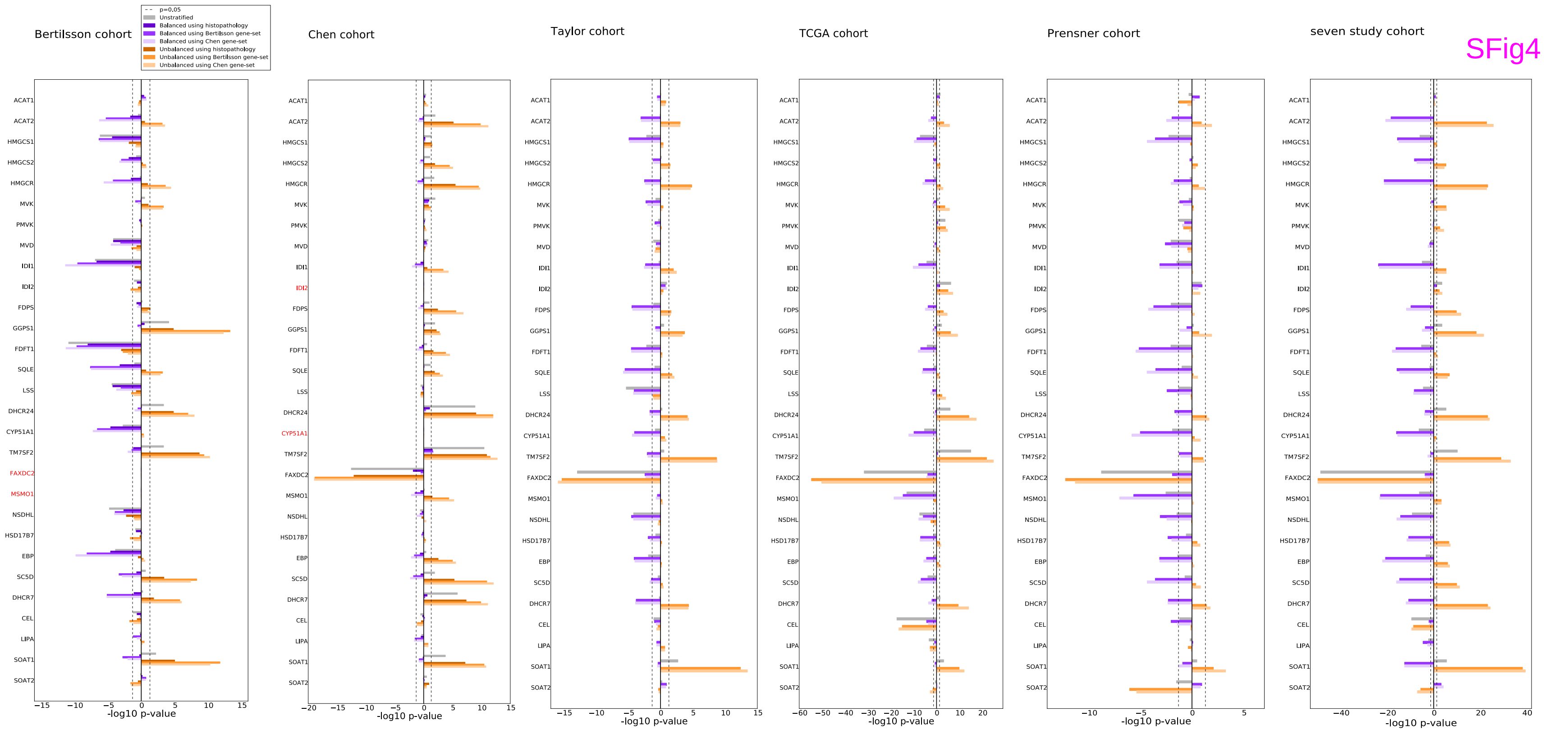

SFig4
p=0.05
Unstratified
Balanced using histopathology
Balanced using Bertilsson gene-set
Balanced using Chen gene-set
Unbalanced using histopathology
Unbalanced using Bertilsson gene-set
Unbalanced using Chen gene-set
Bertilsson cohort
Chen cohort
Taylor cohort
TCGA cohort
Prensner cohort
seven study cohort
ACAT1
ACAT2
HMGCS1
HMGCS2
HMGCR
MVK
PMVK
MVD
IDI1
IDI2
FDPS
GGPS1
FDFT1
SQLE
LSS
DHCR24
CYP51A1
TM7SF2
FAXDC2
MSMO1
NSDHL
HSD17B7
EBP
SC5D
DHCR7
CEL
LIPA
SOAT1
SOAT2
-log10 p-value

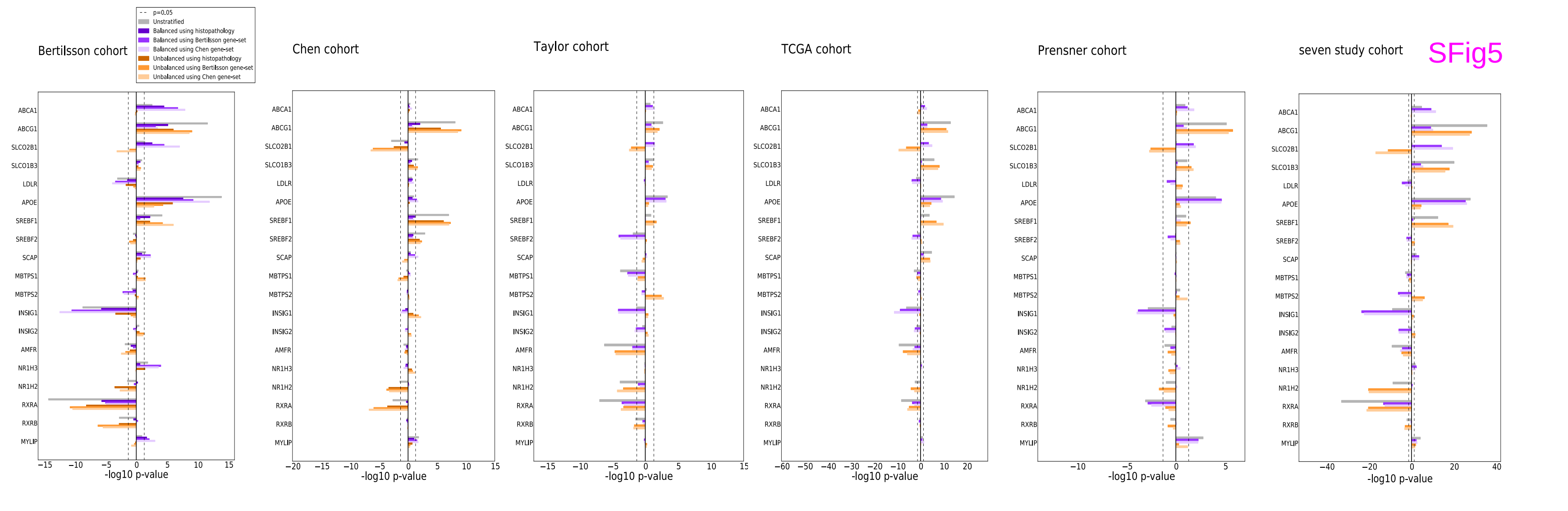
SFig5
Bertilsson cohort
Chen cohort
Taylor cohort
TCGA cohort
Prensner cohort
seven study cohort
p=0.05
Unstratified
Balanced using histopathology
Balanced using Bertilsson gene-set
Balanced using Chen gene-set
Unbalanced using histopathology
Unbalanced using Bertilsson gene-set
Unbalanced using Chen gene-set
ABCA1
ABCG1
SLCO2B1
SLCO1B3
LDLR
APOE
SREBF1
SREBF2
SCAP
MBTPS1
MBTPS2
INSIG1
INSIG2
AMFR
NR1H3
NR1H2
RXRA
RXRB
MYLIP
-log10 p-value

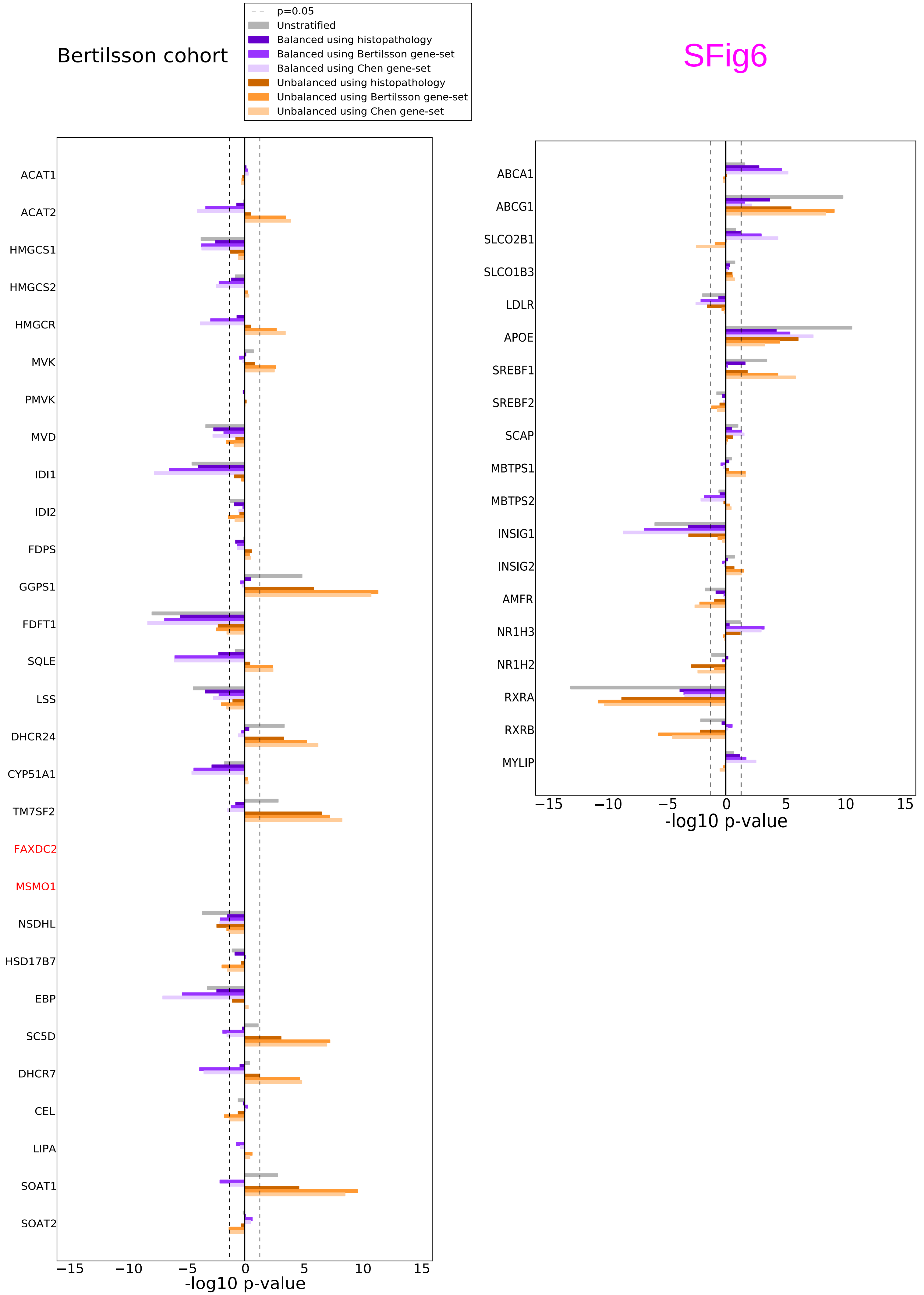
Bertilsson cohort
p=0.05
Unstratified
Balanced using histopathology
Balanced using Bertilsson gene-set
Balanced using Chen gene-set
Unbalanced using histopathology
Unbalanced using Bertilsson gene-set
Unbalanced using Chen gene-set
ACAT1
ACAT2
HMGCS1
HMGCS2
HMGCR
MVK
PMVK
MVD
IDI1
IDI2
FDPS
GGPS1
FDFT1
SQLE
LSS
DHCR24
CYP51A1
TM7SF2
FAXDC2
MSMO1
NSDHL
HSD17B7
EBP
SC5D
DHCR7
CEL
LIPA
SOAT1
SOAT2
−15 −10 −5 0 5 10 15
-log10 p-value
ABCA1
ABCG1
SLCO2B1
SLCO1B3
LDLR
APOE
SREBF1
SREBF2
SCAP
MBTPS1
MBTPS2
INSIG1
INSIG2
AMFR
NR1H3
NR1H2
RXRA
RXRB
MYLIP
−15 −10 −5 0 5 10 15
-log10 p-value

SFig6

STab1: GSEA score stabilities using various numbers of the top ranked stroma genes in the gene-sets from *Bertilsson* and *Chen*.

GSEA score stabilities using various subsets of the top scoring stroma genes in gene-sets from *Bertilsson* and *Chen*. Ten subsets of 100, 150, 200, 250, 300, 350, 400, 450, 500 and 1000 genes were chosen from each gene-set, and the GSEA score using each subset was normalized to a 0-100 range for comparison. For the *Bertilsson* and *Chen* datasets, which also had available histopathology, the stability of the prediction of percentage normal stroma content was also assessed by a linear regression model. Both the 9527 shared genes in the *five-study-cohort*, and the 4804 shared genes in the *seven-study-cohort* were used in independent GSEA calculations. The mean standard deviation of normalized GSEA scores over all samples, as well as the maximum standard deviation was calculated for each cohort. Generally the prediction displayed high stability, both over the selection of gene-set size and the number of genes used for GSEA calculations. Note that the normalization of 0-100 only serves as an approximation of predicted stroma content, and that true predictions of percentage stroma can only be performed in the *Bertilsson* and *Chen* cohorts where histopathology is available.

Cohort	Bertilsson gene-set							
	9527 shared genes (5 cohorts)				4804 shared genes (7 cohorts)			
	GSEA score		Predicted stroma		GSEA score		Predicted stroma	
	Mean std	Max std	Mean std	Max std	Mean std	Max std	Mean std	Max std
Bertilsson	2.53	3.70	1.05%	2.56%	2.47	4.44	1.27%	3.10%
Chen	2.51	3.98	1.20%	2.65%	2.62	5.16	1.31%	2.69%
Taylor	2.11	4.45			2.58	5.45		
TCGA	1.41	3.40			1.70	3.57		
Prensner	1.87	3.98			2.14	7.41		
Sboner					2.98	7.76		
Erho					3.56	7.74		

Cohort	Chen gene-set							
	9527 shared genes (5 cohorts)				4804 shared genes (7 cohorts)			
	GSEA score		Predicted stroma		GSEA score		Predicted stroma	
	Mean std	Max std	Mean std	Max std	Mean std	Max std	Mean std	Max std
Bertilsson	2.13	5.09	1.13%	2.86%	2.04	4.69	1.22%	2.58%
Chen	1.76	3.77	0.86%	2.00%	1.80	3.72	1.07%	2.22%
Taylor	1.65	3.60			2.41	5.23		
TCGA	1.55	3.77			2.07	4.59		
Prensner	1.51	3.16			1.60	3.85		
Sboner					2.29	5.92		
Erho					2.47	5.62		

STab2: Number of cancer/normal samples and average Gleason score in *balanced* and *unbalanced* datasets from all cohorts.

Cohort	N. samples Cancer / Normal Balanced	N. samples Cancer / Normal Unbalanced	Gleason Score Balanced / Unbalanced Histopathology	Gleason Score Balanced / Unbalanced Bertilsson gene-set	Gleason Score Balanced / Unbalanced Chen gene-set
**Bertilsson**	58 / 20	58 / 20	7.00 / 7.31	7.28 / 7.00	7.47 / 6.81
**Chen**	32 / 35	33 / 36	6.75 / 6.93	6.81 / 6.88	6.84 / 6.85
**Taylor**	65 / 14	66 / 15	NA	6.49 / 6.53	6.52 / 6.50
**TCGA**	248 / 26	249 / 26	NA	7.58 / 7.55	7.66 / 7.47
**Prensner**	39 / 19	39 / 19	NA	NA	NA
**Sboner**	140 / 0	141 / 0	NA	7.31 / 7.10	7.26 / 7.14
**Erho**	272 / 0	273 / 0	NA	7.43 / 7.66	7.45 / 7.64
